# The perfect birth: a content analysis of midwives’ posts about birth on Instagram

**DOI:** 10.1186/s12884-023-05706-2

**Published:** 2023-06-07

**Authors:** Anna Marsh, Vanora A. Hundley, Ann Luce, Yana Richens

**Affiliations:** 1 Centre for Midwifery Maternal & Perinatal Health, Bournemouth University, Bournemouth Gateway Building, St Pauls Lane, Bournemouth, BH8 8GP UK; 2 Elizabeth Garrett Anderson Unit, University College London Hospitals NHS Foundation Trust, 25 Grafton Way, London, WC1E 6DB UK; 3 Department of Journalism and Communication, Bournemouth University, Weymouth House, Talbot Campus, Fern Barrow, Poole, BH12 5BB UK; 4 Liverpool Women’s NHS Foundation Trust, Crown Street, Liverpool, L8 7SS UK

**Keywords:** Social Media, Maternity, Midwifery, Birth, Social Media Influencer

## Abstract

**Background:**

There is limited research into how midwives use social media within their professional role. Small pilot studies have explored the introduction of social media into maternity practice and teaching but there is little evidence around how midwives use social media professionally. This is important as 89% of pregnant women turn to social media for advice during pregnancy, and how midwives use social media could be influencing women, their perception of birth and their decision making.

**Methods:**

**Aim:**

To analyse how popular midwives portray birth on the social media platform Instagram.

This is an observational mixed methods study using content analysis. Five ‘popular’ midwives from each country (UK, New Zealand, USA and Australia) were identified and their posts about birth collated from a one-year period (2020–21). Images/videos were then coded. Descriptive statistics enabled comparison of the posts by country. Categorisation was used to analyse and understand the content.

**Results:**

The study identified 917 posts from the 20 midwives’ accounts, containing 1216 images/videos, with most coming from USA (*n* = 466), and UK (*n* = 239), Australia (*n* = 205) and New Zealand (*n* = 7) respectively. Images/videos were categorised into ‘Birth Positivity’, ‘Humour’, ‘Education’, ‘Birth Story’ and ‘Advertisement’. Midwives’ portrayals of birth represented a greater proportion of vaginal births, waterbirths and homebirths than known national birth statistics.

The most popular midwives identified mainly had private businesses (*n* = 17). Both the midwives and women portrayed in images were primarily white, demonstrating a disproportionate representation.

**Conclusion:**

There is a small midwifery presence on Instagram that is not representative of the broader profession, or the current picture of midwifery care. This paper is the first study to explore how midwives are using the popular social media platform Instagram to portray birth. It provides insight into how midwives post an un-medicalised, low risk representation of birth. Further research is recommended to explore midwives’ motivation behind their posts, and how pregnant and postnatal women engage with social media.

## Introduction

Fear and anxiety related to birth has increased on a global scale [1], and maternity services are beginning to look more broadly for the cause of this increase. Birth has been popularised by ‘fly on the wall’ television programmes such as ‘One Born Every Minute’ and ‘One Born Every Minute USA’ [2], but these programmes are known to be overdramatic and overly medicalised for entertainment effect [3]. The global culture of birth is likely to be affected by other medians and influences. One hypothesis is the effect of social media.

Women are using social media as a source of advice and support during childbirth [4, 5], with one study finding that as many as 89% of pregnant women turn to social media for information and advice [4]. At present, research around the content to which pregnant women are exposed on social media is limited. For first time mothers, birth is an unknown, ‘behind closed doors’ event, which can lead to fear and anxiety [6]. Television has been explored as a method of providing visual insight through popular programmes, but often found over-medicalisation depictions of birth designed to entertain which can lead in turn to an unrealistic expectation of birth [3, 7]. However similar research has not been conducted within social media. Studies have shown that social media can affect individuals’ physical and mental health [8, 9], lifestyle choices [10, 11] and even influence buying preferences [12, 13], so it is quite possible that it is having an influence on women’s choices and expectations about birth. Despite this, there is very little research within this field.

Evidence suggests that health discussion and promotion on social media contain high levels of incorrect information [14]. Despite this, internationally there is limited research into the topic or disclosure of professional usage of social media by midwives [15]. Reasons for this are voiced as fear of professional retribution or uncertainty of what to say [15–17]. However, small studies have begun to emerge within maternity services demonstrating positive effects for both women and midwives when using social media and that it can be used as a supportive community and platform for knowledge sharing [15, 18].

In 2020, 3.6 billion people used social media globally, with the most popular platform being Facebook with 2.9 billion active users [19]. However, over the past few years the platform Instagram has risen in popularity with the younger generations, to the point where it is now the most popular with people of childbearing age [20]. In 2022, Instagram had a total of one billion active users, 86% of which were under the age of 45 [21]. The platform is largely visual based, with users sharing an image or video with a short caption with which other users around the world to ‘like’ or ‘comment’.

Whilst anyone can have an account, Instagram has facilitated the uprising of ‘Instagram Influencers’ who are a group of popular account holders who use their status and engagement with their followers to market products and achieve celebrity status [22, 23]. The definition of a social media ‘Influencer’ varies, but for this study the definition used was *‘prominent social media users who accumulated a dedicated following by crafting an authentic online persona’* [23]. Within the sphere of public health, concerning connections have already been made between Influencers and the impact on health promotion and eating disorders [24]. However, on a broader level, targeted public health promotions have had some success on Instagram [25, 26], suggesting that with further research there is a potential for positive change. Despite this, there is no research into the role of popular midwives, women or other stakeholders and their influence as ‘influencers’ on women during their childbirth journey.

If social media can strongly influence an individual’s health and their choices, then it could be argued that there is a role for the midwife in steering individuals’ understanding, beliefs and choices around birth. This study aims to analyse how popular, or ‘influencer’ midwives portray birth on the social media platform Instagram.

## Methods

This study was an observational mixed methods study using media content analysis to analyse data from Instagram. The primary objective was to analyse how midwives post about birth on the social media platform Instagram. As healthcare systems vary significantly across the world, influences from different systems could be reaching pregnant women via global platforms. Therefore, this study looks to explore how midwives post about birth, with comparisons between the UK, New Zealand, USA and Australia.

The study used media content analysis, a method frequently used within media and communication studies [27]. Health researchers may be more familiar with content analysis as a qualitative data analysis tool as opposed to research method [28, 29]. However, within the field of media it is a fundamental method used to ‘analyse data within a specific context in view of the meanings someone – a group or a culture – attributes to them’ [27]. Content analysis is beginning to emerge as an interdisciplinary method, and was found as the method most common used when exploring the use of other social medias within health research [30]. Media content analysis is a sub-set of content analysis, and is most popularly used because of its flexibility, ability to include both qualitative and quantitative data and robust theoretical underpinning to understanding themes and concepts [31, 32]. First introduced in 1927 by Harold Lasswell as an systematic method for analysing mass media in the context of propaganda [33], it is now an established and popular method of analysing dynamic media data [34]. Considering the contemporary, innovative nature of this research, this method lends itself to both the fields of health and media used within this study.

### Ethics

Prior to commencement of this study, ethics approval was achieved through Bournemouth University Ethics Committee.

Social media content is already freely available for use within the public domain, however consideration was taken to anonymise Instagram account information as best practice.

### Account selection

In consideration of the concept of ‘Instagram influencers’ [22, 23], a strategy was created to identify ‘popular’ midwifery accounts. This would enable further understanding of the information and content from accounts that childbearing women are most likely to be exposed to. Accounts from the professional bodies within each country were considered, however their follower count significantly lower than some midwives’ and therefore their reach considered less. As well as this, their accounts may be supported by Communications teams or advisors, and therefore their posts not made by midwives exclusively. Therefore, popular midwives, or ‘midwifery influencers’ were chosen. Five Instagram accounts of midwives were chosen from four countries: United Kingdom (UK), Australia (Aus), New Zealand (NZ) and the United States of America (USA). These countries were chosen as a group of high-income westernised, English-speaking countries with different healthcare cultures.

A pilot study was undertaken to ensure the strategy collected midwives’ accounts that would provide sufficient data. This led to a purposive strategy of selecting midwives identified as ‘Influencers’ by:

- Reviewing blogs and websites of ‘the best midwives to follow’
- Using a generic search engine to search for ‘ < country > midwife Instagram’ and reviewing the first 20 results
- Identifying accounts mentioned in the last 20 posts by each country’s professional body’s Instagram account or events pages.

Inclusion and exclusion criteria are shown below:

- Inclusion criteria:
  - ‘Midwife’ of ‘Labour RN’ declared on account
  - Recent posts (5 or more posts within the last two years)
  - Accounts not ‘Private’, and therefore publicly available
- Exclusion criteria
  - Nurses, doulas or other allied professionals
  - Not active on account in two years

Accounts were then reviewed to ensure that they met inclusion criteria and collated. If more than five accounts qualified per country, purposeful selection was made of the accounts with the most Followers.

### Data collection

Data were collected using the datascraping tool PhantomBuster, which was chosen due to its ease of use and the applicability of the information it scrapes. All posts related to birth were identified by a researcher throughout the period 1/9/2020 to 31/08/2021 to accommodate for fluctuations throughout the year, and the URLs collated. URLs were then inputted into PhantomBuster to produce databases of raw data on each post including post URL, account holder, captions, number of likes and comments and date published.

Due to the pioneering nature of this study, no coding sheets or data collection tools existed related to Instagram and birth. Therefore a manual data collection tool was developed including codes based on De Benedictis, Johnson [35] and elements from the NHS birth plan template [36] as a tool used by women to make decisions about birth. Further to this, inducive coding was used to group commonly arising topics that were not included in either code sheet. It is recognised that codes such as ethnicity could be subjective, so broader codes such as ‘white’, ‘black’ or ‘other minority ethnic’ were included to reduce inaccuracies. Where posts overlapped several codes, dominant coding, or hegemonic coding [37], was used to allocated the code deemed most prominent or appropriate. This was most relevant in the categorisation as outlined below, in which some posts may have been posted to share a birth story for example, but also featured an advert for the midwife who was present. Additionally, some videos portrayed various elements of labour such as several stages or positions. In these instances, posts were allocated to the most prevalent or represented category. As the primary researcher is a midwife, professional knowledge and experience was used to aid interpretation. Two researchers trialled this tool prior to use, and then each post was reviewed and inputted into the tool.

An element of the coding including Categorisation. Categorisation is an element of Discourse Analysis often used within Journalism and Communication research. Categories were made by considering Potter and Wetherell [38] as a method of analysis of discursive construct and understanding content. In this instance, the method was used to analyse the overarching sentiment of the post, opening opportunity for more in-depth review. Categories were developed initially by data immersion and consideration of the ‘sentiment’ of the post. Categories were developed, proposed to the research team and discussed. Final categories were agreed as ‘Education’, ‘Birth Positivity’, ‘Birth Story’, ‘Advertisement’ and ‘Humour’ (Table 1). To ensure validity and reduce the subjectiveness, the categories were included in the intercoder reliability test.

**Table 1:**
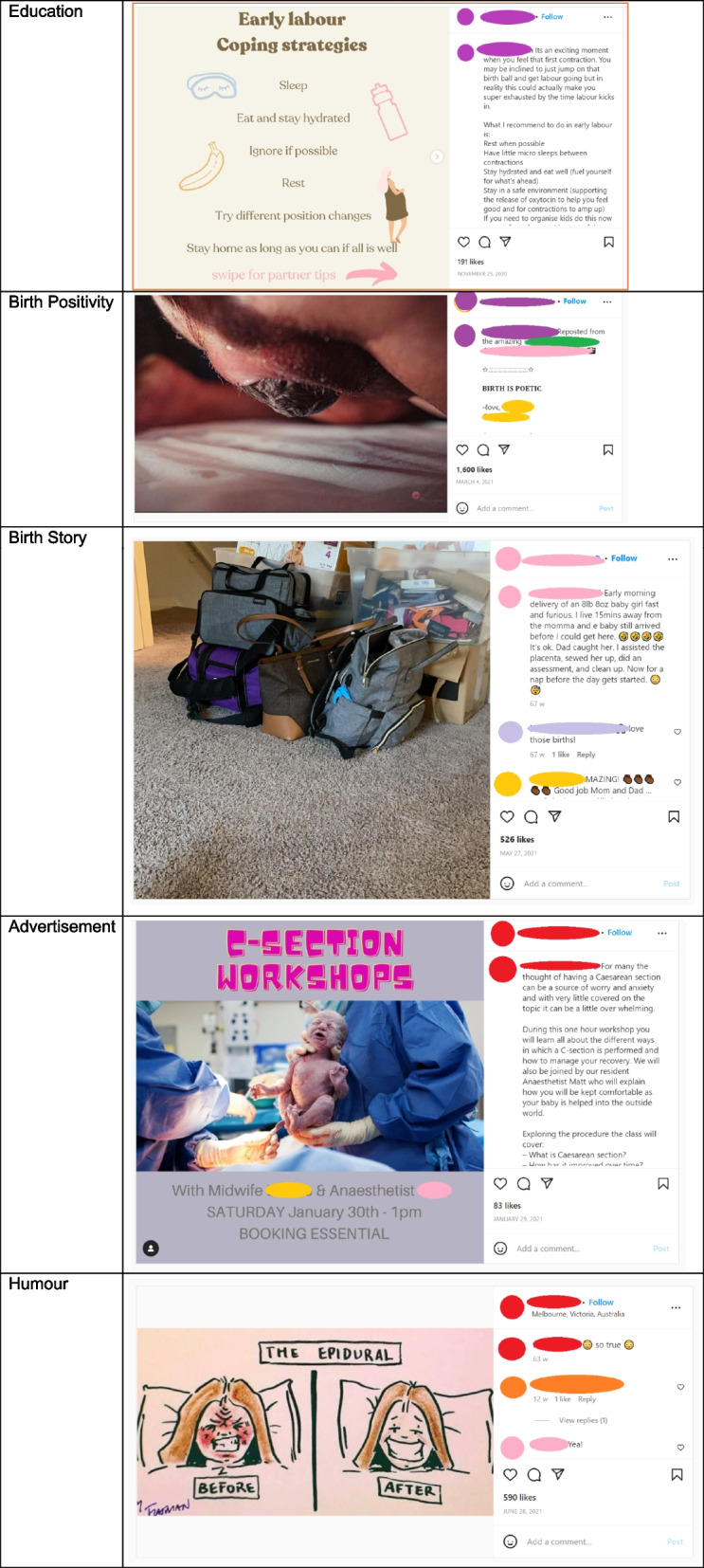
Examples posts within each category

Intercoder reliability was ensured by one researcher separately coding 5% of posts (*n* = 60), randomly selected using an online random number generator. In line with Miles and Huberman [39], an acceptable level of agreement was assumed as 80%. Researchers discussed any significant discrepancies between the posts, and the primary researcher’s coding was used where codes were not agreed. Table 6 outlines the findings.

### Data analysis

Data were analysed using content analysis and simple nominal descriptive statistics to enable comparison of the four countries (Tables 2, 3 and 4). Descriptive statistics involves the presentation and organisation of numerical and graphical data [40]. A sub-analysis focussed specifically on the photographic representation of labour and birth in light of increasing fear of birth. This included reviewing posts in the context of the online NHS Birth Plan [36], which proposes considerations that a woman should make prior to birth.

## Results

Five midwifery accounts from each country (UK, Australia, New Zealand and the USA) were identified (Table 2). A total of 918 posts related to birth were identified from the 20 midwives, including 1218 images or videos. Instagram posts consist of at least 1 image or video with a text caption. Some posts contain multiple images or videos, with a maximum of ten per post.

### Midwifery demographics

Midwives from the USA had the most followers (*n* = 130,836) and post numbers (*n* = 1731) on average (Table 2). The majority of midwives found through the search strategy were of white ethnicity (90%). One midwife from the UK and one midwife from the USA were from other ethnic groups.

**Table 2:** Midwifery demographics

	**UK**		**USA**		**NZ**		**Aus**	
Mean Number of Followers	50,489		130,836		5736		36,837	
Total Number of all-time Posts	551		1731		839		1279	
Ethnicity of Account holder								
White	4	*80%*	4	*80%*	5	*100%*	5	*100%*
Black	0	*0%*	1	*20%*	0	*0%*	0	*0%*
Other Minority Ethnic	1	*20%*	0	*0%*	0	*0%*	0	*0%*
Account holders with associated businesses	4	*80%*	5	*100%*	3	*60%*	5	*100%*
Antenatal and/or Postnatal Education Classes	4	80%	5	*100%*	3	*60%*	5	*100%*
Yoga/Fitness	1	*20%*	1	*20%*	0	*0%*	0	*0%*
Book/E-Book	2	*40%*	1	*20%*	0	*0%*	1	*20%*
Resources for healthcare professionals	1	*20%*	1	*20%*	0	*0%*	0	*0%*
Private Midwifery Care*	0	*0%*	2	*40%*	1	*20%*	1	*20%*

Footnote: *In New Zealand, community midwives work through small practices and are reimbursed through the national health system

Most midwives had private businesses associated with their accounts (*n* = 17). Of those businesses, 100% (*n* = 17) provided Antenatal and/or Postnatal Education, 24% (*n* = 4) sold a book and 24% (*n* = 4) offered direct midwifery care. Two businesses (12%) offered resources for healthcare professionals.

### Post engagement

Table 3 outlines the posts’ content and engagement. The USA received the highest mean ‘Likes’ per post (*n* = 3721), with UK second (*n* = 1369), Australia third (*n* = 580) and NZ fourth (*n* = 323). The USA received the highest mean number of ‘Comments’ (*n* = 123), with the UK and Australia receiving similar numbers at *n* = 60 and *n* = 58 respectively. Posts from New Zealand received the lowest number of ‘Comments’ with an average of 13 per post.

**Table 3:** Post content

	**UK**		**USA**		**NZ**		**Aus**	
	n	%	n	%	n	%	N	%
**Number of images/videos related to birth (n)**	**263**		**667**		**7**		**281**	
Number of posts	239		466		7		206	
Number of images	228		460		7		263	
Number of videos	35		207		0		18	
Category of Image/Video								
Birth Positivity	41	*16%*	144	*22%*	4	*57%*	71	*25%*
Humour	2	*1%*	20	*3%*	0	*0%*	2	*1%*
Educational	178	*68%*	215	*32%*	2	*29%*	172	*61%*
Birth Story	24	9%	277	42%	1	14%	13	5%
Advertisement	18	7%	11	2%	0	0%	23	8%
Engagement								
Total number of likes	327,283		1,733,865		2263		119,547	
Mean number of likes per post	1369		3721		323		580	
Total number of comments	14,429		57,194		89		11,856	
Mean number of comments per post	60		123		13		58	
Photographic Images/Videos of Labouring Women								
Images and Videos	87	*33%*	466	*70%*	7	*100%*	107	*38%*
Images	78	*30%*	323	*48%*	7	*100%*	98	*35%*
Videos	9	*3%*	143	*21%*	0	*0%*	9	*3%*

### Post content

Midwives from the USA posted the most posts about birth (*n* = 466), followed by the UK (*n* = 239), Australia (*n* = 206) and New Zealand (*n* = 7) (Table 3). As posts can contain more than one image or video, the total numbers of images or videos about birth within these posts were: USA (*n* = 667), UK (*n* = 263), Australia (*n* = 281) and New Zealand (*n* = 7). In Australia, New Zealand and the UK, images outweighed videos significantly, but in the USA videos represented nearly a third of all content.

Images/videos were most likely to be ‘Educational’ in the UK (68%) and Australia (61%) (Table 3). A further analysis of these Educational posts was undertaken and reported outside the remit of this paper [41]. In the USA, most images/videos were Birth Stories (42%), whereas this only represented a small proportion of the UK (9%), Australia (5%) and New Zealand (14%) images/videos. In New Zealand ‘Birth Positivity’ (57%) represented the highest category. ‘Humour’ was the category that appeared least frequently in each country but was included to ensure full representation and as the posts included did not fit meaningfully into other categories.

### Photographic representation of labour and birth

Of the total images/videos about birth, New Zealand posted the largest proportion of photographic representations (100%), followed by the USA (70%), Australia (38%) and finally the UK (33%) (Table 3). Table 4 describes the stages of labour and the positions that women adopted. In Table 5 the place of birth and the type of birth are reported.

**Table 4:** Photographic images/videos of women in labour

	**UK**		**USA**		**NZ**		**Aus**	
	n	%	n	%	n	%	N	%
	*n* = 87	*33%*	*n* = 466	*70%*	*n* = 7	*100%*	*n* = 107	*38%*
Stage of Labour								
Latent Phase	1	*1%*	0	*0%*	0	*0%*	0	*0%*
First Stage	19	*22%*	69	*15%*	1	*14%*	27	*25%*
Second Stage	19	*22%*	177	*38%*	0	*0%*	24	*22%*
Third Stage	22	*25%*	164	*35%*	0	*0%*	29	*27%*
After Third Stage	25	*29%*	55	*12%*	2	*29%*	27	*25%*
Not Clear	1	*1%*	1	*1%*	0	*0%*	0	*0%*
Position in 1^st^ stage	*n* = 19		*n* = 69		*n* = 1		*n* = 27	
Recumbent/Semi-Recumbent	0	*0%*	1	*1%*	0	*0%*	0	*0%*
Sitting/Squatting/Kneeling	15	*79%*	44	*64%*	1	*100%*	18	*67%*
Standing	4	*21%*	23	*33%*	0	*0%*	8	*30%*
Theatre bed/LSCS	0	*0%*	0	*0%*	0	*0%*	0	*0%*
Lying Down	0	*0%*	1	*1%*	0	*0%*	0	*0%*
Lithotomy	0	*0%*	0	*0%*	0	*0%*	0	*0%*
N/A	0	*0%*	0	*0%*	0	*0%*	1	*4%*
Analgesia visible	*n* = 87	*33%*	*n* = 466	*70%*	*n* = 7	*100%*	*n* = 107	*38%*
Nil Visible	57	*66%*	239	*51%*	4	*57%*	70	*65%*
Epidural	7	*8%*	1	*1%*	1	*14%*	1	*1%*
Entonox	2	*2%*	0	*0%*	0	*0%*	6	*6%*
Hydrotherapy	21	*24%*	226	*48%*	2	*29%*	30	*28%*
Cannula Visible?								
Yes	11	*13%*	17	*4%*	3	*43%*	7	*7%*
No	76	*87%*	449	*96%*	4	*57%*	100	*93%*
CTG Visible?								
Yes	11	*13%*	7	*2%*	1	*14%*	1	*1%*
No	76	*87%*	459	*98%*	6	*86%*	106	*99%*
Ethnicity of Labouring/Birthing Woman								
White	65	*75%*	295	*63%*	6	*86%*	91	*85%*
Black	8	*9%*	119	*26%*	0	*0%*	0	*0%*
Minority Ethnic	7	*8%*	32	*7%*	1	*14%*	11	*10%*
Unclear/Unknown	7	*8%*	20	*4%*	0	*0%*	5	*5%*
Professional Present	*n* = 87		*n* = 466		*n* = 7		*n* = 107	
Uniformed healthcare professional	26	*30%*	69	*15%*	0	*0%*	18	*17%*
Non-Uniformed healthcare professional	7	*8%*	116	*25%*	1	*14%*	11	*10%*
Not Clear	0	*0%*	33	*7%*	0	*0%*	11	*10%*
No healthcare professionals in image/video	54	*62%*	248	*53%*	6	*86%*	67	*63%*

In total, 42% of images and videos showed women using a pool for analgesia, with numbers as high as 48% in the USA (Table 4). Overall, 94% of women did not have a cannula visible and 97% did not show a CTG. Women were largely in mobile, upright positions during the 1^st^ stage of labour, with only 2 images demonstrating a women semi-recumbent/recumbent or laying down (2%).

Healthcare professionals were rarely featured in images/videos, with 56% not including a healthcare professional at all. The UK featured the most images with a uniformed healthcare professional visible during birth and labour (30%), whereas fewer from Australia (17%), USA (15%) and New Zealand (0%) featured a healthcare professional.

In all countries, the majority of images/videos of birth were of white women. The USA (26%) and UK (9%) were the only countries where black women were represented.

The representations largely depict an un-medicalised portrayal of birth, with the majority of births across all countries (where mode of birth was clear) being spontaneous vaginal births (75%) (Table 5). Instrumental births were rarely portrayed, with only seven from the USA, one from the UK and none from Australia or New Zealand. The highest proportion of images with caesarean section births was from the UK (21%), followed by Australia (12%), USA (9%) and then New Zealand (0%). Homebirths were portrayed in 65% of images/videos of birth from the USA, but were in only 17% of UK births, 23% in Australian births and 0% in births in New Zealand. The location of birth most represented in the UK (40%) and New Zealand (43%) was clinical labour rooms.

**Table 5:** Photographic images/videos of birth

	**UK**		**USA**		**NZ**		**Aus**	
	n	%	n	%	n	%	N	%
Mode of birth								
Number of images/videos where mode of birth is clear	*n* = 56		*n* = 340		*n* = 2		*n* = 110	
SVD (total)	33	*59%*	304	*89%*	2	*100%*	42	*38%*
SVD on land	15	*27%*	115	*34%*	1	*50%*	24	*22%*
SVD in water	18	*32%*	189	*56%*	1	*50%*	18	*16%*
Instrumental Births (total)	1	*2%*	7	*2%*	0	*0%*	0	*0%*
Kiwi/Ventouse	0	*0%*	7	*2%*	0	*0%*	0	*0%*
Forceps	1	*2%*	0	*0%*	0	*0%*	0	*0%*
LSCS (total)	12	*21%*	29	*9%*	0	*0%*	13	*12%*
Emergency LSCS	5	*9%*	1	*1%*	0	*0%*	0	*0%*
Elective LSCS	0	*0%*	0	*0%*	0	*0%*	1	*1%*
Unknown category of LSCS	7	*13%*	28	*8%*	0	*0%*	12	*11%*
Location of labour/birth	*n* = 87	*33%*	*n* = 466	*70%*	*n* = 7	*100%*	*n* = 107	*38%*
Birth Centre	0	*0%*	10	*2%*	0	*0%*	0	*0%*
Clinical Labour Room	35	*40%*	44	*9%*	3	*43%*	18	*17%*
Home	15	*17%*	302	*65%*	0	*0%*	25	*23%*
Theatre	11	*13%*	27	*6%*	0	*0%*	16	*15%*
None of the above	1	*1%*	2	*1%*	0	*0%*	2	*2%*
Unclear	25	*29%*	81	*17%*	4	*57%*	46	*43%*
Position in 2^nd^ stage	*n* = 19		*n* = 177		*n* = 0		*n* = 24	
Recumbent/Semi-Recumbent	1	*5%*	34	*19%*	0	*0%*	2	*8%*
Sitting/Squatting/Kneeling	11	*58%*	117	*66%*	0	*0%*	11	*46%*
Standing	3	*16%*	9	*5%*	0	*0%*	0	*0%*
Theatre bed/LSCS	1	*5%*	11	*6%*	0	*0%*	6	*25%*
Lying Down	0	*0%*	1	*1%*	0	*0%*	4	*17%*
Lithotomy	2	*11%*	0	*0%*	0	*0%*	0	*0%*
N/A	2	*11%*	5	*3%*	0	*0%*	2	*8%*
Livebirth or Stillbirth?								
No. of images/videos including babies	*n* = 55		*n* = 435		*n* = 6		*n* = 302	
Livebirth	55	*100%*	434	*99%*	6	*100%*	302	*100%*
Stillbirth	0	*0%*	1	*1%*	0	*0%*	0	*0%*
Of photographic images/videos of labour/birth, was it censored by Instagram?								
Yes	3	*3%*	51	*11%*	0	*0%*	2	*2%*
No	84	*97%*	415	*89%*	7	*100%*	105	*98%*

Intercoder reliability tests were undertaken in collaboration with another researcher (VH) (Table 6). All fields met the pre-determined threshold of 80% agreement.

**Table 6:** Intercoder Reliability

Code	Percentage agreement
Category	81.7%
Race of Birthing Person	91.7%
Mode of Birth	88.3%
Stage of Labour	81.7%
Birth Location	90%
Pain Relief	100%
Cannula visible?	100%
CTG visible?	96.7%
Outcome	96.7%
Birth Position (1^st^ stage)	98.3%
Birth Position (2^nd^ stage)	95%
Birth Position (3^rd^ stage)	90%
Birth Professional Present	88.3%

## Discussion

Overall, the midwifery influencers, or ‘most popular’ midwives on Instagram were not representative of the profession and this study suggests that the profession does not have a strong presence on the social media platform Instagram. How they posted about birth, including mode of birth, location of birth and ethnicity of the birthing person, was not representative of known birth statistics in each country, portraying a much lower intervention version of birth.

Unless midwives had a business or something to market then they appear less likely to engage with Instagram, or with considerably less outreach. Even where midwives did engage, their following was small. Some lifestyle Instagram influencers have several million followers [22], yet the midwives with the highest number of followers from the USA only averaged 130,836. Midwives currently have nowhere near the same outreach or impact as other influencers, yet they have many clear public health promotion agendas. Considering that women are using social media for advice in guidance in pregnancy and the postnatal period [4, 42, 43], there is clear space to increase the engagement of midwives and service users on social media, in turn providing the profession with an opportunity to improve communication with women and their families.

The popular midwives included in this study were predominantly white, with only two out of the twenty midwives being from other ethnic backgrounds. Whilst this may be an artefact of the sampling strategy, it is relevant as these midwives had the highest follower count and are therefore likely to reach the most women. This high disproportion is mirrored in their posts of women in labour, of whom 69% were white. Considering that in each country, maternal morbidity and mortality are highest for women from black or minority ethnic backgrounds (including indigenous women) [44–47], clear inequalities are already present for these women across maternity services. This high proportion of white midwives posting about white women could be providing further failures in communication with those known to already be at higher risk.

Representation was not only skewed in terms of ethnicity, but also in the picture of labour and birth depicted. When midwives posted photographic images and videos of women, the focus was on an unmedicalized labour and birth with high rates of vaginal birth and homebirth. Whilst there are risks and benefits for both caesareans and vaginal births and a personalised approach should be taken, it is largely accepted that morbidity and mortality are overall lower for low risk mothers and babies after a vaginal birth [48]. Similarly, homebirths are known to have increased positive outcomes for women’s outcomes and experiences [49, 50]. Despite this, in other areas of the media, such as television, birth was found to be more medicalised and riskier for dramatic effect [3].This therefore could suggest midwives are reclaiming the narrative and posting their perception of ‘the perfect birth’. Considering that women who requested caesarean births cite fear of birth and uncertainty around vaginal birth as key reasons [51], it could also be proposed that midwives are providing visual education and solutions to these concerns with their Instagram posts. Further research is needed into the motivation behind midwives posts, but it appears as though their content represents a more classic, low risk ‘Midwifery model’ [52].

Whilst the focus on more physiological birth may be positive for a midwifery model of care, thought needs to be directed towards how women receive this information and the effects that it has on them. How women receive information is largely unknown within the research. Audience theories, from Media and Communications fields, explore how people respond to information within the media, proposing that this is linked to an individuals’ background, motivation, their passive or active consumption of information and age amongst other factors [53]. Birth is known to be an ‘unseen event’ for which women seek to educate themselves, so it is argued that a picture of physiological birth could be actively or passively skewing their expectations or preferences of birth. More research is clearly needed to explore the effect of social media on women, however considering a woman’s health and experience can be affected by birth expectations not being met [54–57], social media influence could be hypothesised to be linked to a woman’s outcomes.

It is also noteworthy that most influencer midwives had businesses associated with their Instagram accounts (*n* = 17). Whilst this may not be surprising in countries where maternity care is largely private, such as the USA, in countries like the UK and Australia where most women can access free care this is clearly disproportionate. All businesses sold Antenatal Education, which is in line with findings that 77% of women turned to their smartphone for antenatal education [58]. With limited guidance around social media use at national level, midwives have taken this as an opportunity to create their own enterprises. It could be argued that these midwives are using Instagram as a marketing tool. Vrontis, Makrides [22] explored the impact of social influencer marketing on new businesses, finding that whilst an influencer needed to be perceived as credible, sales were also improved by psychological influence on the audience, such as ‘wishful identification’. Applying this to the midwives within the study, to market their businesses the midwives could be posting wishful ‘ideal’ birth images to improve sales, rather than just relying on their credentials alone. This study did not have the resources to interview midwives about their motivation for posting, and this should be explored in the future.

It appears that within the field, research around social media is rarely undertaken by midwives. Of the small amount of published material available around pregnancy or birth on Instagram, none to date is authored by midwives. Most studies were by individuals working in the field of journalism, communication or digital or social media studies [59–65] or other fields such as psychiatry [66], public health specialists [67] or one by an obstetrician [68]. This gap in the research field clearly mirrors the lack of midwifery presence on Instagram reported above; however it leaves the field open to alternative channels and voices to provide information and advice with varying motives and intentions. Why this dearth of evidence or presence exists is largely unknown, but it is clear that headway needs to be made by midwives to create a strong presence on social media and the surrounding research field.

Early research to explore why midwives don’t engage with social media or the broader media has found a common theme of fear of professional retribution for saying or doing the wrong thing [15, 16]. For those midwives who are using Instagram, this study clearly demonstrates that they are not portraying birth representatively and are possibly excluding minority groups from the potential benefits. Therefore, it is proposed that to target both the fear and inaccurate use of social media, training for practising midwives should be implemented.

There are many limitations of this research. First, only midwives who posted using English language were included due to limited resources. It is also relevant that the data scraping and data analysis involved in analysing social media data is extremely time consuming. The use of dominant coding may unintentionally have reinforced existing mainstream discourse, however every attempt was made to provide a balanced narrative. Although only 20 accounts were chosen, the volume of data was significant, and reducing this to the content relevant to this study took a significant amount of time. This limited the sample size. Furthermore, considering that only seven posts were included from New Zealand, it is likely that the selection strategy did not identify some significant midwifery users on Instagram. It is recognised that whilst influencers were chosen to gain insight into the content to which women were most likely to be exposed, it does have potential to limit their representation of the broader profession. Whilst this research does explore the content that midwives posted, it does not explore their intention or motivation when posting, or how women have received the information.

## Conclusion

This is the first study to explore how popular midwives are using the social media platform Instagram. The findings indicate a small midwifery presence that is unlikely to be representative of the broader profession, or the current picture of midwifery care. Midwives posted a largely un-medicalised portray of birth, and the influence of this on women and their expectations of birth needs further exploration. Given the potential for social media to influence individuals’ understanding, beliefs and choices around birth, this is an area that the midwifery profession needs to develop. Further research is recommended to explore midwives’ motivation behind their posts, as well as how pregnant or postnatal women receive information through social media. Tailored training packages for midwives and student midwives to empower them to use social media and refine their current usage may be helpful.

## Data Availability

All data is in the public domain and available from Instagram using the link: https://www.instagram.com/. The datasets used and analysed during the current study available from the corresponding author on reasonable request.
